# Supplementary material for: Synergistic effects of proteinaceous pheromone and nitrogen starvation on male gametogenesis in the anisogamous volvocine alga Eudorina
Source: PLoS One. 2025 Nov 21;20(11):e0326066. doi: 10.1371/journal.pone.0326066 (PMC12637917; doi:10.1371/journal.pone.0326066)
Supplement: S1 Fig — (A) Schematic illustrations of representative developmental stages based on the time-course data in (B) and (C). The light–dark condition (16-h light/8-h dark) is indicated by the bar at the bottom. (B) Time course of the total colony number under synchronous culture conditions (see Materials and Methods). One hundred pre-hatching colonies were incubated in nitrogen-rich medium, and colonies were counted every 3 h. Daughter colonies were released by ~6 h (hatching stage), reaching ~250 colonies/mL, and the number then remained constant through the maturation and cleavage stages (~21 h). The second hatching stage began at ~24 h and continued until ~30 h. On average, 83.1% of the initially hatched colonies released next-generation colonies (~3,600 colonies/mL). Each value represents the mean ± SE (n = 3). The male strain used was 2022–1122-EF4-M1.(C) Time course of the cleavage-stage colonies from the same experiment shown in (B). Colonies were classified as cleavage stage if they contained at least one embryo undergoing cell division. Cleavage-stage colonies appeared soon after the dark period began (~18 h), increased until ~27 h, overlapping with the second hatching stage (B), and were completed by ~30 h. Each value represents the mean ± SE. (PDF) [file pone.0326066.s001.pdf]

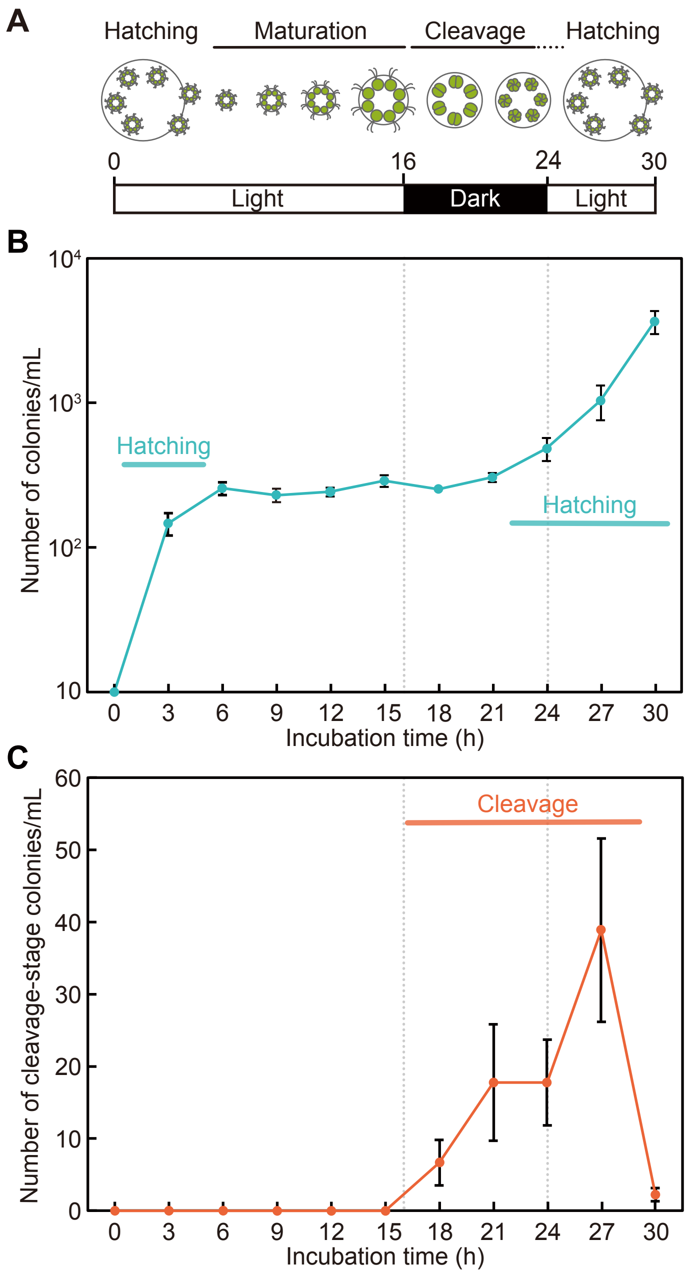

S1 Fig. Quantitative analysis of vegetative colony (VC) development in synchronously cultured *Eudorina.*

(A) Schematic illustrations of representative developmental stages based on the time-course data in **(B)** and **(C)**. The light–dark condition (16-h light/8-h dark) is indicated by the bar at the bottom.

(B) Time course of the total colony number under synchronous culture conditions (see Materials and Methods). One hundred pre-hatching colonies were incubated in nitrogen-rich medium, and colonies were counted every 3 h. Daughter colonies were released by ~6 h (hatching stage), reaching ~250 colonies/mL, and the number then remained constant through the maturation and cleavage stages (~21 h). The second hatching stage began at ~24 h and continued until ~30 h. On average, 83.1% of the initially hatched colonies released next-generation colonies (~3,600 colonies/mL). Each value represents the mean ± SE (n = 3). The male strain used was 2022-1122-EF4-M1.

(C) Time course of the cleavage-stage colonies from the same experiment shown in **(B)**. Colonies were classified as cleavage stage if they contained at least one embryo undergoing cell division. Cleavage-stage colonies appeared soon after the dark period began (~18 h), increased until ~27 h, overlapping with the second hatching stage **(B)**, and were completed by ~30 h. Each value represents the mean ± SE.
