# Supplementary material for: Synergistic effects of proteinaceous pheromone and nitrogen starvation on male gametogenesis in the anisogamous volvocine alga Eudorina
Source: PLoS One. 2025 Nov 21;20(11):e0326066. doi: 10.1371/journal.pone.0326066 (PMC12637917; doi:10.1371/journal.pone.0326066)
Supplement: S2 Fig — In Volvox, the vegetative cycle (yellow-shaded area) takes ~48 h under a 16-h light/8-h dark cycle, compared to ~24 h in Eudorina. This longer duration is due to the prolonged expansion phase, during which both the parental colony and its enclosed daughter colonies expand for ~24 h after inversion. When gonidia sense the sex pheromone 6–8 h before the cleavage stage, they undergo modified type of embryogenesis to form a colony containing androgonidia (blue-shaded area). Subsequently, the androgonidia divide and form SPs through incomplete inversion. (PDF) [file pone.0326066.s002.pdf]

***Volovox carteri* male**

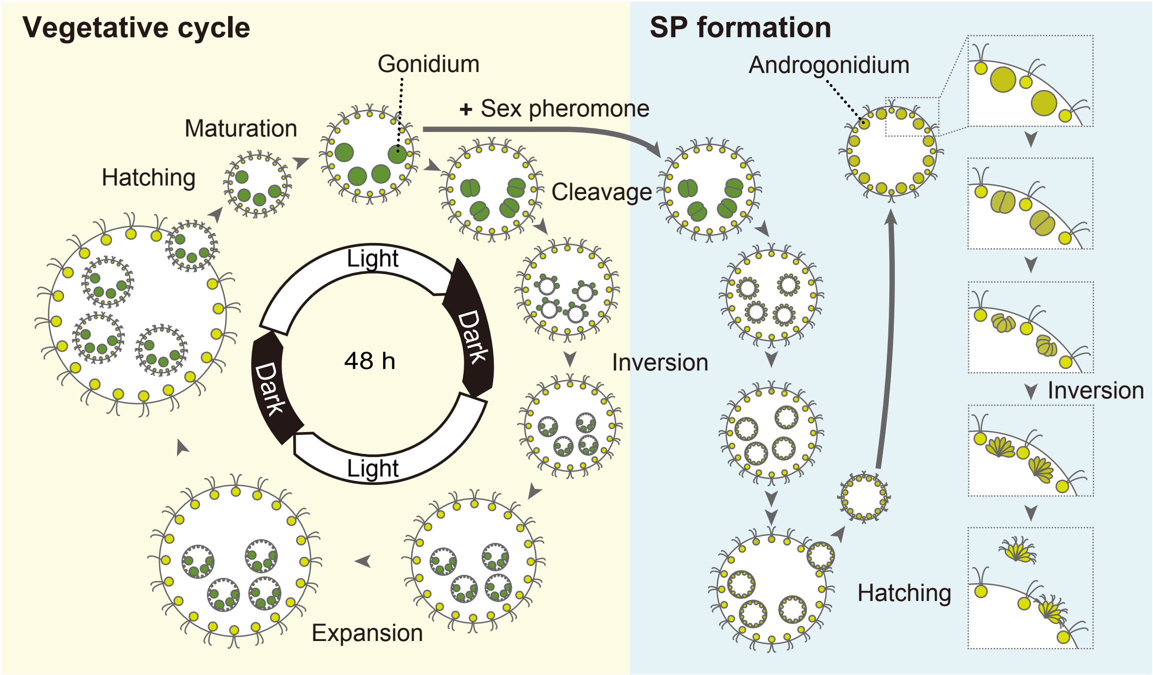


**S2 Fig. Schematic representation of the vegetative cycle and sperm packet (SP) formation in the *Volvox carteri* male strain (for comparison).**

In *Volvox*, the vegetative cycle (yellow-shaded area) takes ~48 h under a 16-h light/8-h dark cycle, compared to ~24 h in *Eudorina*. This longer duration is due to the prolonged expansion phase, during which both the parental colony and its enclosed daughter colonies expand for ~24 h after inversion. When gonidia sense the sex pheromone 6–8 h before the cleavage stage, they undergo modified type of embryogenesis to form a colony containing androgonidia (blue-shaded area). Subsequently, the androgonidia divide and form SPs through incomplete inversion.
