# Supplementary material for: Synergistic effects of proteinaceous pheromone and nitrogen starvation on male gametogenesis in the anisogamous volvocine alga Eudorina
Source: PLoS One. 2025 Nov 21;20(11):e0326066. doi: 10.1371/journal.pone.0326066 (PMC12637917; doi:10.1371/journal.pone.0326066)
Supplement: S3 Fig — Pre-heated pronase was mixed with either mating medium or CM to a final concentration of 200 μg/mL (see Materials and Methods). These mixtures were added to cultures of vegetative male colonies at a final volume of 10% (v/v), and the SP ratios were measured. SP formation exceeded 80% in CM-treated cultures regardless of the presence of heat-treated pronase, while no SPs were formed in cultures without CM, regardless of the presence of heat-treated pronase. These results indicate that heat-treated pronase does not affect the SP formation. Each value represents the mean ± SE from three biological replicates (≥54 colonies in total). The male strain used was 2022–1122-EF4-M1. (PDF) [file pone.0326066.s003.pdf]

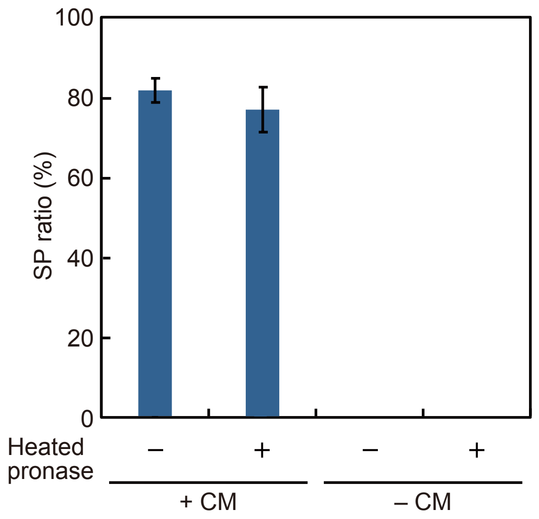

S3 Fig. Effect of heat-treated pronase on SP formation.
Pre-heated pronase was mixed with either mating medium or CM to a final concentration of 200 µg/mL (see Materials and Methods). These mixtures were added to cultures of vegetative male colonies at a final volume of 10% (v/v), and the SP ratios were measured. SP formation exceeded 80% in CM-treated cultures regardless of the presence of heat-treated pronase, while no SPs were formed in cultures without CM, regardless of the presence of heat-treated pronase. These results indicate that heat-treated pronase does not affect the SP formation. Each value represents the mean ± SE from three biological replicates (≥54 colonies in total). The male strain used was 2022-1122-EF4-M1.
