# Supplementary material for: Synergistic effects of proteinaceous pheromone and nitrogen starvation on male gametogenesis in the anisogamous volvocine alga Eudorina
Source: PLoS One. 2025 Nov 21;20(11):e0326066. doi: 10.1371/journal.pone.0326066 (PMC12637917; doi:10.1371/journal.pone.0326066)
Supplement: S4 Fig — The uncropped silver-stained SDS-PAGE gel image corresponding to Fig 4 is shown. Lanes labeled “(NH4)2SO4 fraction #1–4” are not included in Fig 4. These lanes represent ammonium sulfate fractionation samples of CM (#1: 0–40% saturation; #2: 40–60% saturation; #3: 60–80% saturation; #4: final supernatant). They are presented here to show the entire gel but are not relevant to the present study. (PDF) [file pone.0326066.s004.pdf]

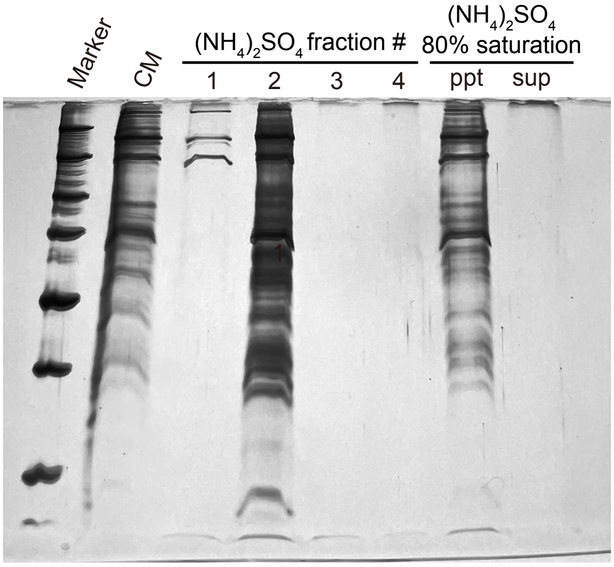

S4 Fig. Raw gel image for Fig 4.

The uncropped silver-stained SDS-PAGE gel image corresponding to Fig 4 is shown. Lanes labeled "$(NH_4)_2SO_4$ fraction #1–4" are not included in Fig 4. These lanes represent ammonium sulfate fractionation samples of CM (#1: 0–40% saturation; #2: 40–60% saturation; #3: 60–80% saturation; #4: final supernatant). They are presented here to show the entire gel but are not relevant to the present study.
