# Supplementary material for: Synergistic effects of proteinaceous pheromone and nitrogen starvation on male gametogenesis in the anisogamous volvocine alga Eudorina
Source: PLoS One. 2025 Nov 21;20(11):e0326066. doi: 10.1371/journal.pone.0326066 (PMC12637917; doi:10.1371/journal.pone.0326066)
Supplement: S1 Table — (PDF) [file pone.0326066.s006.pdf]

S1 Table. Composition of VTAC and mating medium based on [S1, S2].

Components	VTAC (nitrogen-rich)	Mating medium (nitrogen-deficient)
$Ca(NO_3)_2\cdot 4H_2O$	11.78 mg	–
$CaCl_2\cdot H_2O$	–	7.5 mg
β-Na_2 glycerophosphate·$5H_2O$	5 mg	5 mg
$MgSO_4\cdot 7H_2O$	4 mg	4 mg
KCl	5 mg	–
Glycylglycine	50 mg	50 mg
Na acetate·$3H_2O$	20 mg	20 mg
Vitamin B_{12}	0.01 µg	0.01 µg
Biotin	0.01 µg	0.01 µg
Thiamine HCl	1 µg	1 µg
P IV metals*	0.3 mL	0.3 mL
Distilled water	99.7 mL	99.7 mL

The pH of VTAC and mating medium was adjusted to 7.5 and 7.8, respectively, with 0.1 N NaOH.

*P IV metals (in 100 mL distilled water): $Na_2EDTA\cdot 2H_2O$, 100 mg; $FeCl_3\cdot 6H_2O$, 19.6 mg; $MnCl_2\cdot 4H_2O$, 3.6 mg; $ZnSO_4\cdot 7H_2O$, 2.2 mg; $CoCl_2\cdot 6H_2O$, 0.4 mg; Na_2MoO_4, 0.25 mg

Supplementary references.

S1. Nozaki H, Kuroiwa H, Mita T, Kuroiwa T. *Pleodorina japonica* sp. nov. (Volvocales, Chlorophyta) with bacteria-like endosymbionts. Phycologia. 1989;28: 252-267.

S2. Kasai F, Kawachi M, Erata M, Mori F, Yumoto K, Sato M, et al. NIES-collection list of strains, 8th edition. Jpn J Phycol. 2009;57: 1-350.
